# Outcomes of Minimally Invasive Glaucoma Surgery (MIGS) in Glaucoma Patients With Coexisting Cataract: A Systematic Review and Meta-Analysis

**DOI:** 10.7759/cureus.77007

**Published:** 2025-01-06

**Authors:** Saad Bidiwala, Dost Jabarkhyl, Kaim Bidiwala

**Affiliations:** 1 General Medicine, Royal Free National Health Service (NHS) Foundation Trust, London, GBR; 2 General Medicine, Luton and Dunstable University Hospital, London, GBR; 3 Emergency Medicine, King's College Hospital, London, GBR

**Keywords:** coexisting cataract, complex glaucoma and cataract surgeries, glaucoma treatment, minimally invasive glaucoma surgery (migs), reductions in intraocular pressure

## Abstract

Glaucoma and cataracts frequently occur together in elderly populations, demanding combined surgical therapeutic strategies. Therefore, this study aimed to assess the clinical outcomes of minimally invasive glaucoma surgery (MIGS) in patients with coexisting open-angle glaucoma (OAG) and cataracts by adopting a meta-analysis research approach. The current meta-analysis followed the Preferred Reporting Items for Systematic Review and Meta-Analysis (PRISMA) guidelines in selecting and screening the studies. A computer-based search of the PubMed, EMBASE, and Cochrane Library databases was carried out using the last search up to November 2024. The quality of the studies included in this review was evaluated by methodological quality using the Joanna Briggs Institute (JBI) checklist while the risk bias of the included randomized controlled trials (RCTs) was assessed using Cochrane Library tools. All statistical analyses were performed using Review Manager (RevMan) version 5.4.0 (The Cochrane Collaboration). Seven studies (five cohort studies and two RCTs) involving 669 eyes of 651 patients were included. Pooled analysis showed that MIGS combined with cataract surgery significantly reduced intraocular pressure (IOP) compared to cataract surgery alone (mean difference: 1.58 mmHg, 95% CI: 0.30 to 2.87, p<0.00001). Additionally, MIGS decreased postoperative medication use (mean difference: -0.79, 95% CI: -1.28 to -0.30, p<0.0001). However, significant heterogeneity was observed (I^2^=85-100%), likely due to variations in study designs, patient characteristics, and surgical techniques. These findings indicate that MIGS is a reasonable approach to decreasing IOP and reducing glaucoma medications in patients with cataracts and OAG. Due to the small size of the incision, it might be useful for individuals who are older and would still like to get the surgery done but with minimum surgery required. However, the durability and safety of several MIGS procedures have not been consistently determined by different techniques and long-term assessments of MIGS need to be evaluated.

## Introduction and background

Glaucoma is reported as a major cause of irreversible blindness that affects 1% to 8% of the overall population [1]. It is estimated that 76 million population (aged between 40 and 60 years) experience primary open-angle glaucoma (POAG), the most common form of the disease. Approximately 4.5 million people with POAG have moderate to severe visual impairment, while 3.2 million have been suffering from blindness globally [2]. The number of people with POAG is projected to rise to 111.8 million in 2040 from the present as a result of the rise in the aging population [3]. It is a progressive ophthalmic disorder, characterized by optic neuropathy (damage to the nerve fiber layer and optic nerve) and acuity loss that can be triggered by high intraocular pressure (IOP) or alternative mechanisms [4]. Cataract is another cause of visual impairment that can frequently coexist with glaucoma in the elderly population due to their shared association with aging. Cataract surgery, which involves replacing the cloudy lens with an intraocular lens (IOL), is a common treatment strategy and can also result in a modest reduction in IOP [5]. 

While the main treatment strategies for glaucoma are generally aimed at managing glaucomatous damage and lowering IOP [6], initial approaches often involve topical therapies, such as latanoprost, bimatoprost, and travoprost, as part of ocular hypotensive strategies [7].

In individuals with excessive IOP, topical ocular hypotensive medicine may delay or avoid POAG, although ocular surface damage and compliance among patients are significant challenges with medical care. Additionally, low tolerability and low compliance with these treatment options can lead to treatment failure. After the failure of the first treatment line, glaucoma surgeries help to prevent visional loss by elevated IOP [8].

Surgical interventions are considered when topical medicines or other treatments (such as laser) are unable to effectively lower IOP. Among surgical approaches for glaucoma, trabeculectomy, ab externo filtration, incisional surgery, and tube shunt surgery are common. However, these ophthalmological measures are accompanied by a high incidence of complications and prolonged healing times [9]. For instance, being the gold standard for surgically treating glaucoma, trabeculectomy is a technically challenging treatment that may proceed poorly because of scarring, generated astigmatism, subsequent cataracts, and reduced quality of life from bleb-related foreign body sensations [10].

However, recent advancements in the treatment of POAG have introduced various devices different from topical medications and incisional surgery. After five years of follow-up, about 50% of glaucoma patients treated with these implants experience failure contributing to increased rates of reoperation. Consequently, these devices are improved by developing their shape, biomaterials, and drainage techniques, which are now called micro-invasive glaucoma surgeries. Currently available micro-invasive glaucoma surgeries are Hydrus Micro-Stent [11], XEN (XEN gel stent) [12], CyPass Micro-Stent [13], and iStent [14]. 

During the last few years, minimally invasive glaucoma surgery (MIGS) has been reported as a less invasive and safer alternative to traditional glaucoma procedures. These devices are designed to target the pathophysiological process to reduce the IOP and enhance the trabecular outflow through a subconjunctival shunt, to avoid the trabecular meshwork, and augment the uveoscleral outflow via suprachoroidal routes [15]. MIGS procedures are developed to reduce IOP by improving the eye’s natural outflow pathways and preventing tissue disruptions via the use of microscopic incisions as compared to conventional surgery [16]. Compared to traditional surgery, these devices are preferred because they are safer, with fewer seriously dangerous complications, and better medium recovery time for patients with mild to moderate glaucoma. Furthermore, individuals who have both cataracts and glaucoma may experience both vision recovery and IOP reduction with combined cataract surgery and MIGS.

As the world's population ages, more people may require treatment for both glaucoma and cataracts, which makes evaluating combination surgical techniques a top concern. Despite the increasing geriatric population, there is an ongoing dispute related to the long-term efficacy of MIGS for the treatment of open-angle glaucoma (OAG) among patients with coexisting cataract. According to some researchers, MIGS may provide only slight advantages over cataract surgery alone [17,18], even if other studies have shown positive results in terms of lowering IOP and reducing reliance on glaucoma drugs. Additionally, there is confusion regarding the optimal course of action for patients with coexisting diseases because the results of various MIGS techniques in combination with cataract surgery have not been properly studied. Thus, this study is designed to evaluate the clinical outcomes of commercially available MIGS devices among OAG patients with coexisting cataract through the adoption of a meta-analysis research approach.

## Review

Methods

Search Design

The Preferred Reporting Items for Systematic Review and Meta-Analysis (PRISMA) guidelines [19] were applied to conduct this meta-analysis evaluating the clinical outcomes of MIGS in glaucoma patients with coexisting cataract. This study was a meta-analysis of already published randomized controlled trials (RCTs), so there was no need for additional ethical review.

*Population, Intervention, Comparison, and Outcome *(*PICO) Framework*

The PICO model was used to design research questions. For meta-analysis, the model provided PICO questions according to the above-mentioned research aims as follows:

Population: Patients with coexisting glaucoma and cataract

Intervention: MIGS; iStent, Trabectome, Hydrus Micro-Stent, Kahook Dual Blade, and other related techniques

Comparison: Cataract surgery alone or surgical treatments for glaucoma

Outcomes: Reduction in IOP post-surgery, the number of glaucoma medications required after surgery

Search Strategy

The study followed PRISMA guidelines to systematically select and screen research articles aligned with our study aims. Data were extracted from three electronic databases: PubMed, EMBASE, and Cochrane Library. The search included all available records in these databases from inception to November 2024.

A combination of MeSH terms and free-text keywords was used to ensure comprehensive retrieval of relevant studies. The search strategy included terms such as: (“minimally invasive glaucoma surgery” OR “MIGS” OR “minimal invasive surgery”) AND (“cataract” OR “visual impairment” OR “blindness” OR “glaucoma” OR “open-angle glaucoma” OR “OAG”) AND (“lowered intraocular pressure” OR “IOP” OR “use of glaucoma medications”). Additionally, the reference lists of all relevant systematic reviews and meta-analyses were examined to identify further eligible studies.

Inclusion and Exclusion Criteria

The eligibility criteria helped in selecting and screening research articles after searching research articles from electronic databases. Only those studies that met the following inclusion criteria were included: (i) Studies analyzing population having glaucoma coexisting with cataract; (ii) Studies analyzing the impacts of MIGS; (iii) Studies tracking the outcomes related to reductions in IOP and several medications used after surgery; (iv) Studies published as cohort studies, RCTs, and observational studies; and (v) Studies with full text available and published in the English language.

The following studies were excluded: (i) Studies analyzing population with glaucoma or cataracts alone; (ii) Studies involving the impacts of incision surgeries and trabeculectomy; (iii) Studies discussing other outcomes rather than the above-mentioned ones; (iv) Studies based on systematic review, meta-analysis, comprehensive reviews, narrative reviews, and editorials; and (v) Studies published in other languages other than English and non-full text papers. 

Data Extraction and Study Outcomes

Two independent reviewers extracted the data to put in the pre-specified table. The data related to demographic information such as authors, year of study, country, study population with mean age, study groups, study design, study follow-up and primary outcomes of reductions in IOP, and number of medications as post-surgery outcomes were extracted. Discrepancies were resolved by consulting a third reviewer.

Risk of Bias Assessment

The Cochrane Library tool was used to assess the risk bias from the included RCTs. The quality of included studies based on Cochrane guidelines was assessed for six aspects of study risk bias: allocation concealment, blinding of participants, selection bias, blinding of outcomes, selective reporting, and other biases. Every included study was assessed for risk of bias, and the risk of bias of each study was assigned to low risk, unclear, and high risk [20]. Furthermore, the Joanna Briggs Institute (JBI) critical appraisal tools were employed to evaluate the methodological quality of these identified cohort studies for this meta-analysis. The critical assessment of the included cohort studies or empirical studies, as well as their approach to addressing and minimizing bias, was presented using the JBI critical assessment instrument. Standardized critical appraisal tools are employed by JBI to evaluate the potential for various biases that may arise in quantitative research. Based on the methodology of studies, there are JBI-standardised appraisal instruments suitable for JBI reviews of efficacy [21]. 

Statistical Analysis

Review Manager (RevMan) version 5.4.0 (The Cochrane Collaboration) was used to conduct all statistical analyses. The pooled analysis of data was performed for studies with potential heterogeneity by using random-effects models. A p-value of <0.05 was considered statistically significant. The effect sizes were shown as mean differences for continuous outcomes such as reductions in IOP and odds ratio for complication rates, number of medications used, and visual acuity. Heterogeneity was evaluated using the I^2^ statistic; I^2^ values above 50% indicated significant heterogeneity.

Results

Search Results

**Figure 1:**
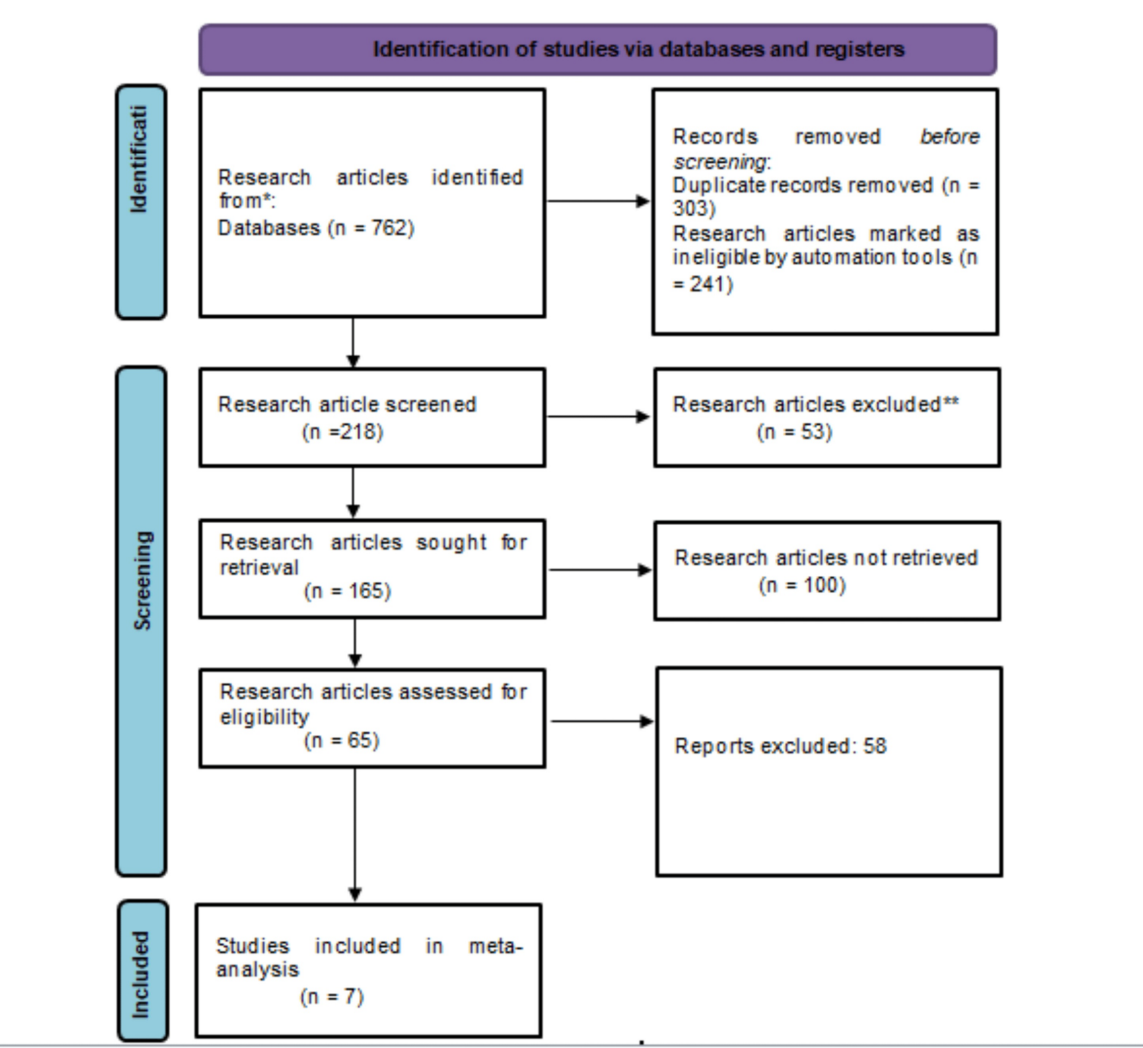
PRISMA flowchart of screening and selection process of research studies PRISMA: Preferred Reporting Items for Systematic Review and Meta-Analysis The figure was created by the authors.

In this meta-analysis, the selection and screening of research articles related to the study's aim was completed by following the PRISMA guidelines. From the three prescribed electronic databases, about 762 research articles were extracted after the application of the search strategy. The eligibility criteria were applied to only 165 articles and seven studies were included in the final pooled analysis, as shown in Figure 1. 

Characteristics of Included Studies

Our study analyzed seven research articles (five retrospective cohort studies and two RCTs) and 669 eyes among 651 OAG patients with coexisting cataract to evaluate the clinical outcomes of MIGS by using a meta-analysis approach. The main characteristics of selected studies for analysis have been presented in Table 1. All included studies were retrospective or prospective observational studies and randomized clinical trials published between 2000 and 2024. The sample size of included studies ranged from 45 to 240 elderly from OAG populations with coexisting cataract. The mean age of elderly patients varied from 64.9 to 74.4 years. The follow-up period in these included studies varied from six months to 10 years.

**Table 1:** Characteristics of included studies PEA: phacoemulsification and aspiration, IOP: intraocular pressure, MIGS: minimally invasive glaucoma surgery, OAG: open-angle glaucoma, T: treatment, P: placebo, IOL: intraocular lens, KDB: Kahook Dual Blade, RCT: randomized controlled trial

Study	Country	Study population (mean age)	Study groups	Study design	Study follow-up	MIGS used	Reductions in IOP (mmHg)	Number of medication
Hayakawa et al. 2024 [17]	Japan	159 eyes of subjects with OAG and cataracts (74.3 years)	T: 95 eyes received MIGS with PEA + IOL, P: 64 eyes received µLot-phaco group (PEA + IOL)	Retrospective cohort study	6 months	Intraocular lens insertion with pharma emulsification	T: 0.18, P: 0.9	
Chen et al. 2021 [22]	USA	55 eyes of 55 adults with OAG and cataracts (70.76 years)	T: 35 iStent eyes, 7 iStent inject eyes, and 13 KDB	Retrospective cohort study	12 months	KDB, iStent, or iStent inject	T: 3.70 ± 4.50, P: 0.15 ± 0.5	T: 1.06 ± 0.32, P: 1.67 ± 0.98
Oberfeld et al. 2024 [23]	USA	71 eyes of 71 patients (72.46 years)	T: 34 received combined MIGS, P: 37 in control	Retrospective cohort study	12 months	MIGS	T: 3.6 (2.4), P: 2.8 (2.2)	T: 1.3 (1.5), P: 2.4 (1.7)
Chang et al. 2021 [24]	USA	45 eyes of 45 adults (74.4 years)	T: 45 received MIGS	Retrospective study	2.5 years	iStent injection or KDB	T: 1.4 (0.2), P: 0.17 (0.3)	T: 1.5 (1.1), P: 2.0 (1.1)
Samuelson et al. 2011 [25]	USA	240 eyes (73 years)	T: 111 received iStent with cataract surgery, P: 122 received cataract surgery only	Prospective RCT	12 months	iStent implantation	T: 4.2 (1.3), P: 0.91 (0.12)	
Fea et al. 2010 [26]	Italy	36 patients (64.9 years)	T: 12 received phacoemulsification with iStent implantation, P: 24 received phacoemulsification alone	Prospective RCT	15 months	Phacoemulsification with iStent implantation	T: 3.1 (1.4), P: 1.6 (1.9)	T: 0.4 ± 0.7, P: 1.3 ± 1.0
Neuhann et al. 2024 [27]	USA	63 eyes of 45 patients	T: 60 received iStent MIGS	Retrospective cohort study	10 years	iStent trabecular micro-bypass stent implantation	T: 3.53 ± 1.2, P: 1.98 ± 0.13	T: 0.45 ± 0.76, P: 1.83 ± 1.03

Quality Assessment

As mentioned above, the JBI tool was utilized for five included cohort cross-sectional studies and empirical studies on the clinical outcomes of MIGS in glaucoma patients with coexisting cataract. The quality assessment as per the JBI checklist is given in Table 2.

**Table 2:** Quality assessment of included studies using JBI tool JBI: Joanna Briggs Institute, N: no, Y: yes, N/A: not applicable, UN: unclear

Questions	Hayakawa et al. 2024 [17]	Chen et al. 2021 [22]	Oberfeld et al. 2024 [23]	Chang et al. 2021 [24]	Neuhann et al. 2024 [27]
Were the two study groups recruited from the same population?	Y	N	Y	Y	N/A
Were the exposures measured similarly to the study population's exposed and unexposed groups?	Y	Y	N	Y	Y
Was the exposure measured in a validly and reliably?	Y	N	Y	N	Y
Were confounding factors identified?	N	Y	N/A	Y	N/A
Were strategies to deal with confounding factors stated?	Y	N	Y	N/A	N
Was the follow-up time reported and sufficient to be long enough for outcomes to occur?	N	Y	N/A	N	N/A
Was appropriate statistical analysis used?	N	Y	N/A	UN	N/A
Was the follow-up completed, and if not, were the reasons why it was not completed explained and investigated?	Y	UN	N	Y	N/A
Were the results accurately and consistently measured?	Y	N/A	Y	N	Y
Did the participants or groups have access to the results at the beginning of the study?	N	Y	N/A	Y	N/A
Were strategies to report the incomplete follow-up used?	N	Y	N/A	Y	N/A

Risk of Bias Assessment

**Figure 2:**
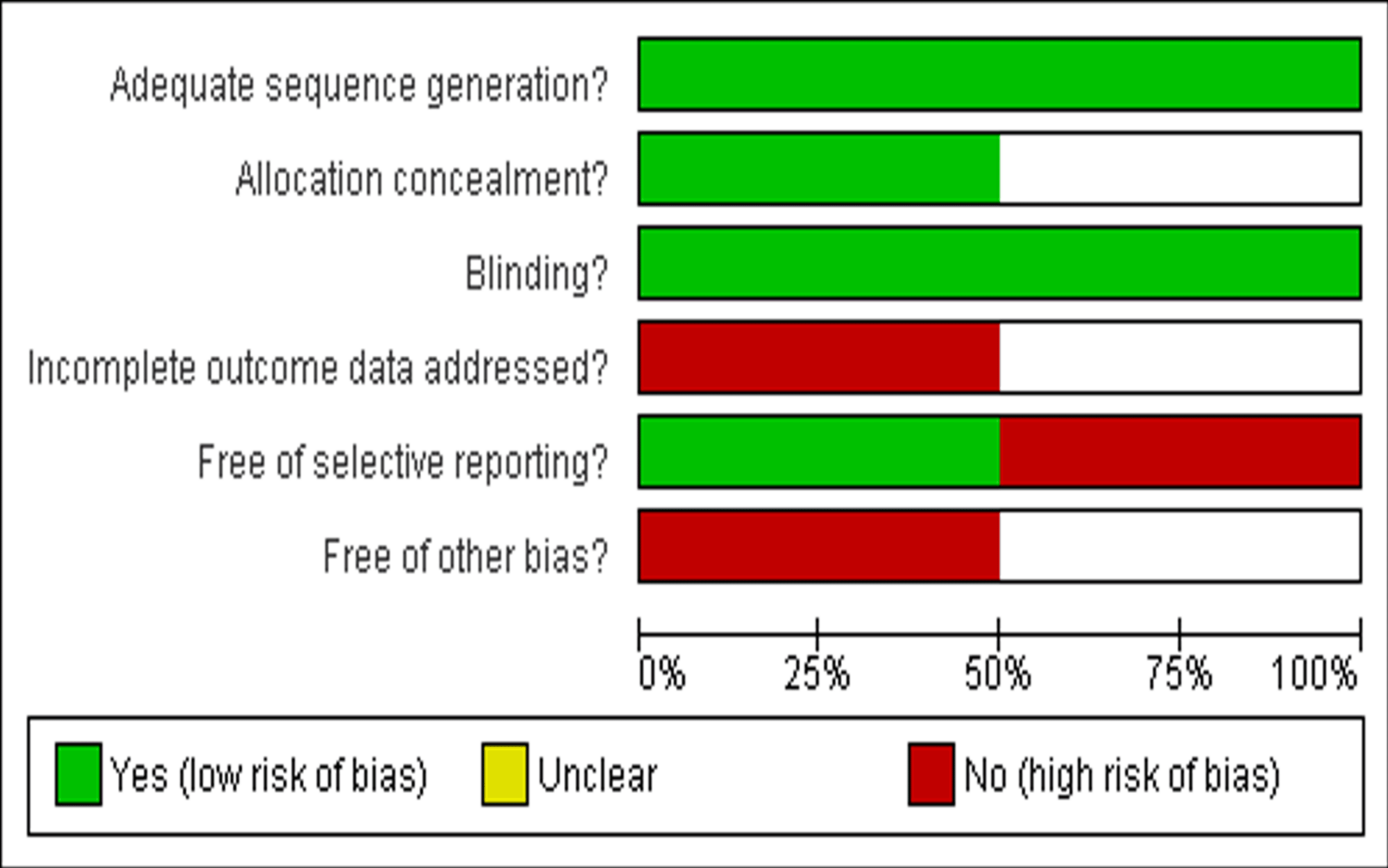
Risk of bias assessment of included studies The figure was created by the authors using Review Manager (RevMan) version 5.4.0 [25,26]

**Figure 3:**
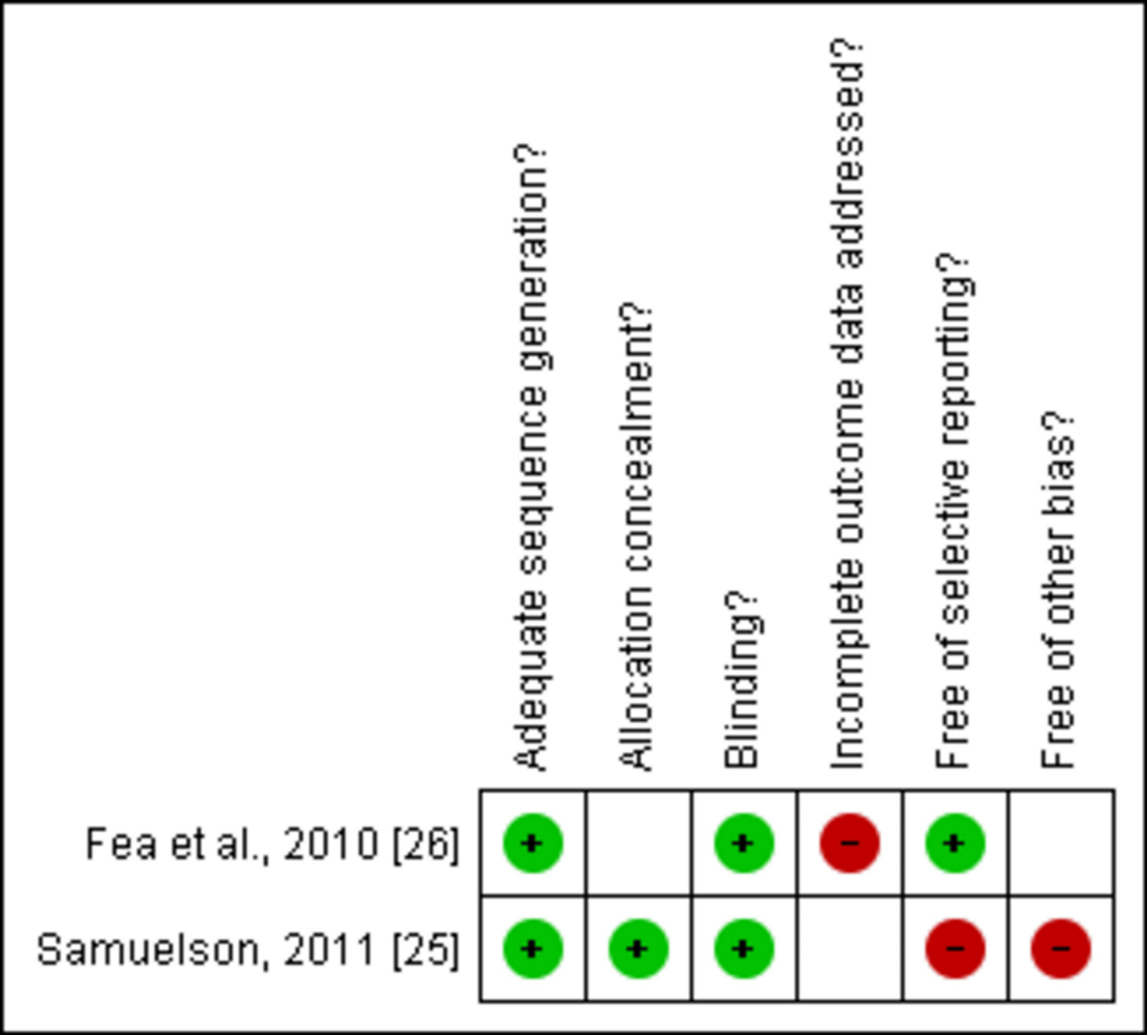
Risk of bias summary of each included RCT RCT: randomized controlled trial The figure was created by the authors using Review Manager (RevMan) version 5.4.0

Concerning the sources of bias, the Cochrane tool was used for the risk bias assessment of the two RCTs included. Between the two RCTs, both studies were low risk [25,26], as shown in Figures 2-3.

Primary Outcomes

This study collected data related to reductions in IOP among glaucoma patients with coexisting cataract after receiving MIGS as compared to placebo.

Reductions in IOP (mmHg) 

**Figure 4:**
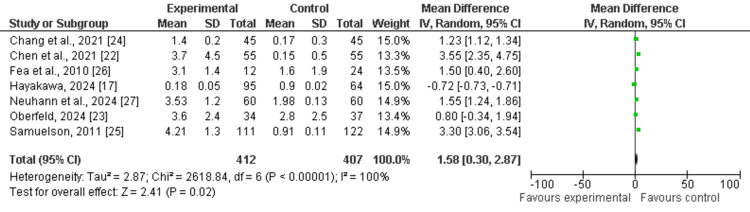
Forest plot of reductions in IOP among experimental and control groups IOP: intraocular pressure The figure was created by the authors using Review Manager (RevMan) version 5.4.0

**Figure 5:**
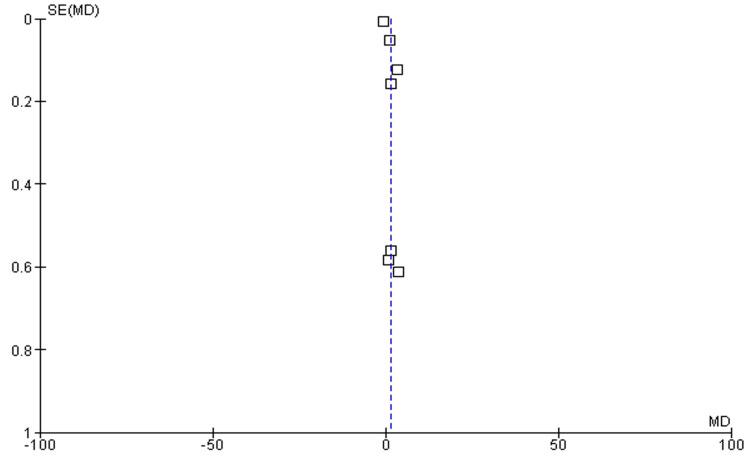
Funnel plot of reductions in IOP among experimental and control groups IOP: intraocular pressure The figure was created by the authors using Review Manager (RevMan) version 5.4.0 [17,22-27]

All included studies have discussed mean reductions in IOP (mmHg) as outcomes among the experimental group (OAG-cataract patients receiving MIGS) compared to the control group. The pooled analysis showed that mean values of reductions in IOP were higher among the experimental group as compared to the control (MD: 1.58 (95% CI: 0.30 to 2.87), p<0.00001) and reported heterogeneity (df=6, I^2^=100), as shown in Figure 4. The funnel plot of symmetrical shape reported low publication bias among the included studies, as shown in Figure 5.

Number of Medication

**Figure 6:**
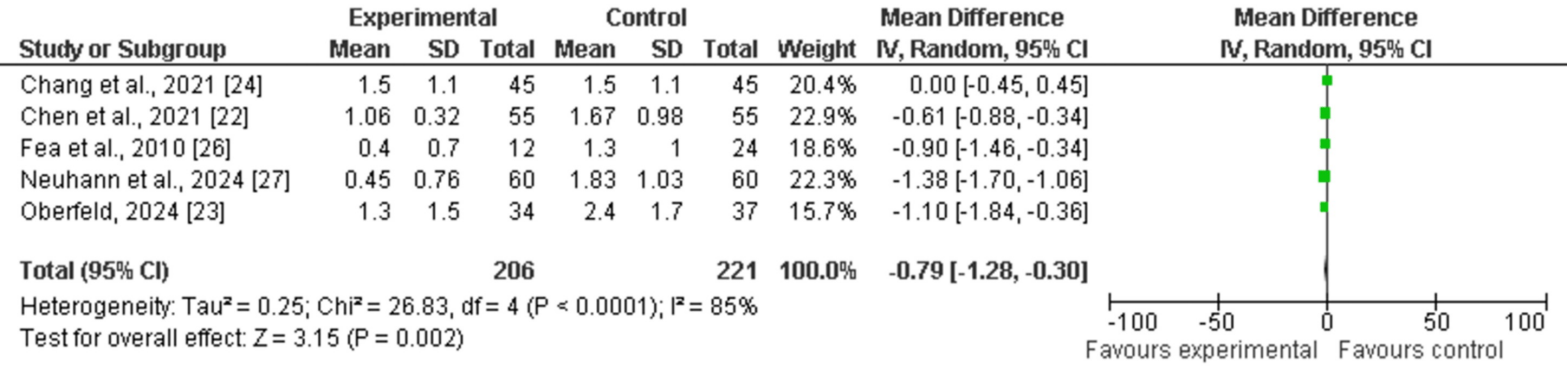
Forest plot of number of medications used postoperatively among experimental and control groups The figure was created by the authors using Review Manager (RevMan) version 5.4.0

**Figure 7:**
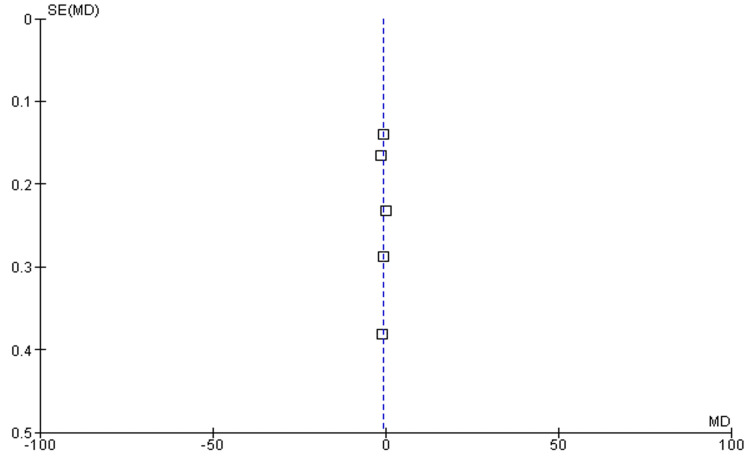
Funnel plot of number of medications used postoperatively among experimental and control groups The figure was created by the authors using Review Manager (RevMan) version 5.4.0 [22-24,26,27]

Among the seven included studies, five have discussed the number of medications used postoperatively as outcomes among the experimental group (OAG-cataract patients receiving MIGS) compared to the control group. The pooled analysis showed that mean values of reductions in IOP were higher among the experimental group as compared to the control (MD: -0.79 (95% CI: -1.28 to -0.30), p<0.0001) and reported heterogeneity (df=6, I^2^=85%), as shown in Figure 6. The funnel plot of symmetrical shape reported low publication bias among included studies, as shown in Figure 7.

Discussion

This study aimed to evaluate the clinical outcomes of MIGS in glaucoma patients with coexisting cataract through the adoption of a meta-analysis research approach. All included studies were published between 2010 and 2024. The mean age of elderly patients varied from 64.9 to 74.4 years. The follow-up period in the included studies varied from six months to 10 years. Through pooled analysis of seven included studies (five cohort studies and two RCTs) and 669 eyes, our study reported that MIGS is an effective management strategy in improving mean values of reductions in IOP (mmHg) and reducing number of medications used postoperatively among OAG patients with coexisting cataract. The pooled analysis showed that mean values of reductions in IOP (mean difference: 1.58 (95% CI: 0.30 to 2.87), p<0.00001) improved, while the number of medications used postoperatively (mean difference: -0.79 (95% CI: -1.28 to -0.30), p<0.0001) reduced among the experimental group receiving MIGS as compared to the control (Figure 4, Figure 6). Since it suggests that MIGS not only decreases IOP but also lessens the burden of long-term drug use, which can improve patient compliance and quality of life, this decrease in medication dependence is clinically significant.

The heterogeneity ranged from 85% to 100%, indicating high and considerable heterogeneity for included studies. The heterogeneity of the pooled estimates may be impacted by the significant variation in study designs, patient demographics, and surgical methods among the studies. Variability in the follow-up period (from six months to 10 years), the kind of MIGS treatment employed, and the severity of glaucoma in various patient cohorts could all contribute to the significant heterogeneity. The symmetrical distributions of studies on the funnel plot showed low publication bias among included studies, enhancing the validity of the results (Figure 5, Figure 7). The checklist developed by JBI was used to evaluate the methodological quality of the included cohort studies (Table 2), and the risk bias of the included RCTs was assessed using the tool from the Cochrane Library.

The results of the present study are in line with the prior investigations that established the benefits and safety of IOP reduction for glaucoma patients with cataract comorbidity [28,29]. Additionally, the clinical outcomes of MIGS related to lowering IOP are uncertain as compared to traditional surgeries such as trabeculectomy that were reported to be more effective in managing IOP, but less safe. Despite this, MIGS is a good choice, especially for individuals with mild to severe glaucoma because of its favorable safety profile, which includes fewer postoperative problems and a quicker recovery. Previous studies have also observed that MIGS can decrease the requirement for medication, with most patients requiring fewer or no drugs following surgery. Given that cataract extraction alone has been demonstrated to occasionally lower IOP by a few millimeters of mercury, this discovery is triggered when MIGS is paired with cataract surgery. This effect is enhanced by MIGS, which enables patients to control their IOP more effectively while taking fewer drugs [30].

The mechanism of action for various MIGS devices, targeting different outflow processes in the eye to lower pressure, contributes to the effectiveness of MIGS procedures, especially iStent and Hydrus Micro-stent, to preserve lower IOP levels over the long term, though the extent of reduced pressure may vary based upon the particular MIGS device utilized. Consequently, individual outcomes may differ based on the device selected and patient variables, even when the combined data show general efficacy.

Major strengths of this study include the use of the meta-analysis approach that synthesized data from studies published between 2010 and 2024. This research question and findings of this study are unique as there is no comprehensive research published on the outcomes of MIGS among glaucoma patients with coexisting cataract. 

Of course, there are certain limitations in the present study despite the strength of the analysis carried out above. Firstly, a small sample of studies was included, which affected the reliability and validity of this study’s outcomes. Secondly, the high heterogeneity of meta-analysis limits the generalization of the results of this study. Determining the overall effectiveness of MIGS is made more difficult by the variation in patient groups, follow-up times, and MIGS equipment. Additionally, even though the pooled IOP decrease is statistically significant, not all patients may benefit clinically from it, especially those with advanced glaucoma who might need more aggressive IOP-lowering techniques. Thirdly, the main focus of our study was to evaluate IOP reduction and medication use, with less emphasis on other important clinical outcomes such as complication rates and visual acuity improvement, compromising the implications of this meta-analysis. Future meta-analyses should aim to try to incorporate these elements in order to offer a more thorough evaluation of the advantages and disadvantages of MIGS in patients with cataracts and glaucoma.

## Conclusions

Overall, the findings of this study suggest that MIGS is an effective option for reducing IOP and minimizing the need for glaucoma medications in patients with coexisting cataract and OAG. The results generally support the use of MIGS as a useful tool in the management of glaucoma, especially in older patients who might benefit from a less invasive surgical approach, apart from the observed heterogeneity. The long-term effectiveness and safety of various MIGS techniques must be further assessed using more consistent methodologies and longer follow-up periods in future research.
